# New Considerations for a Totally Implantable Active Middle Ear Implant

**DOI:** 10.3389/fneur.2021.747887

**Published:** 2021-10-13

**Authors:** Jack Shohet, Jacqueline Bibee

**Affiliations:** Shohet Ear Associates, Orange County, CA, United States

**Keywords:** implants, otology, active middle ear implant, hearing loss, direct drive, speech perception gap

## Abstract

Totally implantable active middle ear implants (AMEI) provide full-time hearing amplification to those with moderate to severe sensorineural hearing loss. While technology in conventional hearing aids (CHA) has advanced greatly, limitations remain for people with active lifestyles, limited vision or dexterity, and hearing aid fit issues. Furthermore, direct-drive properties of AMEI are thought to provide those with inefficient middle ear transfer functions a distinct advantage in delivering prescribed sound to the cochlea, ultimately improving speech understanding with less distortion. AMEI safety, stability, and efficacy outcomes are well documented and fitting strategies continue to improve. Recent studies show how simple aided speech testing can help predict whether a patient struggling with CHA may instead benefit from an AMEI. Totally implantable AMEI continue to be a viable option for patients who cannot or will not utilize traditional hearing aids.

## Introduction

Conventional hearing aids (CHA) are the standard treatment recommendation for 90–95% of people with sensorineural, conductive, and mixed hearing loss (1). However, despite advances in CHA digital technology, hearing aid adoption rate remains low. Recent reports in the U.S. indicate only 34**%** of people with hearing loss wear hearing aids (2) and rates are still <50% in European countries where hearing aids are provided at no cost (3). Of those that do use hearing aids, an average of 8.9 years lapses from candidacy to adoption (4). Several factors have been identified as contributing to limited CHA use and can primarily be grouped into a few categories:

1. Fit: discomfort, otitis externa, occlusion effect, poor retention.
2. Use: limited vision, dexterity, self-efficacy, lost devices, frequent battery changes.
3. Lifestyle: Removal for sleep, water wear, athletic endeavors with heavy perspiration.

Concurrently, risks of untreated hearing loss are becoming clearer. Accelerated cognitive decline and dementia (5), increased fall risk (6) and emergency room visits (7), and mental health challenges (8) have all been linked to hearing loss. Clearly, the need for alternative hearing treatment options is higher than ever.

Active middle ear implants (AMEI) were developed to provide solutions to the above CHA limitations. While four AMEI are commercially available worldwide, only two are totally implantable. This mini review will report the long term device outcomes and updated candidacy considerations for one of these totally implantable AMEI systems.

## The Envoy Esteem

The Envoy Medical Corporation, originally called St. Croix Medical, was founded in 1995 in St. Paul, MN, quickly laying groundwork for a totally implantable hearing aid. What developed was the Envoy Esteem^®^ device, which underwent clinical trials starting in 2000. European CU mark cleared in 2006 and the device was FDA approved in 2010. Uniquely, this AMEI utilizes the tympanic membrane as the natural hearing aid microphone. A piezoelectric sensor on the incus sends ossicular vibration information to a sound processor/battery combination unit, implanted subcutaneously in the postauricular region. The filtered and amplified signal is delivered back to a driver connected to the stapes capitulum, which directly drives the oval window for transduction into the cochlea. This direct drive system contrasts traditional CHA mechanics, which do not account for individual variance in middle ear mechanics affecting the efficiency with which sound reaches the cochlea.

Ongoing device maintenance includes an outpatient surgery on average every 4.9 years (10) to replace the battery. Annual device programming and testing with an Audiologist is recommended. No day-to-day maintenance is required, though some people turn the device volume down or off at night to save battery. Patients receive a remote control for optional volume and programming adjustments.

## Candidacy

Adults with moderate to severe sensorineural hearing loss in one or both ears, ≥40% maximum word understanding, and some experience with CHA are the best candidates for this device. AMEI is not a replacement for those with severe to profound hearing loss needing cochlear implant(s). Additional considerations for the Esteem are anatomical and surgical; Imaging is needed to determine if there is ample middle ear space to insert the device and medical consultation is required to ensure patients are healthy enough to undergo an outpatient surgery lasting on average 4.5 h (11). Contraindications include abnormal middle ear anatomy, recurrent otitis media, mastoiditis, Eustachian tube dysfunction, fluctuating hearing loss, Meniere's disease, retrocochlear pathologies, and disabling tinnitus. Motivated patients are often those who have not tolerated CHA well or desire freedom from routine maintenance of hearing aids i.e. batteries, daily insertion and removal.

## Perioperative Considerations and Surgical Procedure

After establishing audiologic and radiologic candidacy, patients are counseled extensively as to expectations of hearing with a middle ear implant. Existing limitations of current hearing technologies to fully rehabilitate hearing loss must be impressed on the patient who needs to understand that their hearing will not be “normal” and that they will continue to experience difficulties in certain listening situations. The goal of facilitating hearing rehabilitation without an external device and the conveniences, and the conveyed lifestyle benefits (12) are communicated. In addition, the patient is made aware that battery changes will require future surgery and up to 16% of patients will require some type of second surgery other than battery change (10). These revision procedures include transcanal or transmastoid revisions for removal of scar tissue or transducer misalignment or explanation for infection or inadequate benefit. Accommodations are made for the patient to be able to communicate effectively during the 6–8 weeks postoperatively when they will not be able to hear from the implanted ear due to a temporary middle ear serous effusion. Patients are counseled that the ossicular chain is permanently disrupted and although reversible, they are not likely to achieve complete middle ear transmission properties with a reconstructed ossicular chain should the implant have to be removed.

The Esteem surgical procedure is planned as an outpatient procedure unless medically indicated to observe the patient in hospital postoperatively. It requires unique pieces of equipment including a second microscope for laser doppler vibrometry measurements of ossicular movement, a cutting laser and an intracanal microphone assembly to facilitate intraoperative testing. The surgery requires many steps, each of which builds upon earlier ones such that sufficient achievement of each step is required before proceeding. A field clinical engineer performs intraoperative measurements and provides feedback to the surgeon at various steps of the procedure to ensure system integrity. Failure to achieve adequate exposure or transducer alignment can add considerable surgical time. Indeed, a surgeon's first implants often take 6–8 h (10) requiring urinary catheterization. With experience, operative times can be brought down to 2.5–3 h on average (13).

The surgery is done through a postauricular incision large enough to accommodate the amply sized battery/sound processor (SP). A bony trough is drilled through the outer occipitoparietal skull cortex for the SP. A sufficient mastoidectomy is performed to allow the body of the transducers to reside within the mastoid cavity. Adequate exposure requires that bone lateral to the body of the incus is removed and a posterior tympanotomy through the facial recess is performed to facilitate exposure of the lenticular process of the incus and as much of the stapes as is possible to visualize given the anatomic restrictions of the facial nerve and fibrous tympanic annulus.

**Figure 1:**
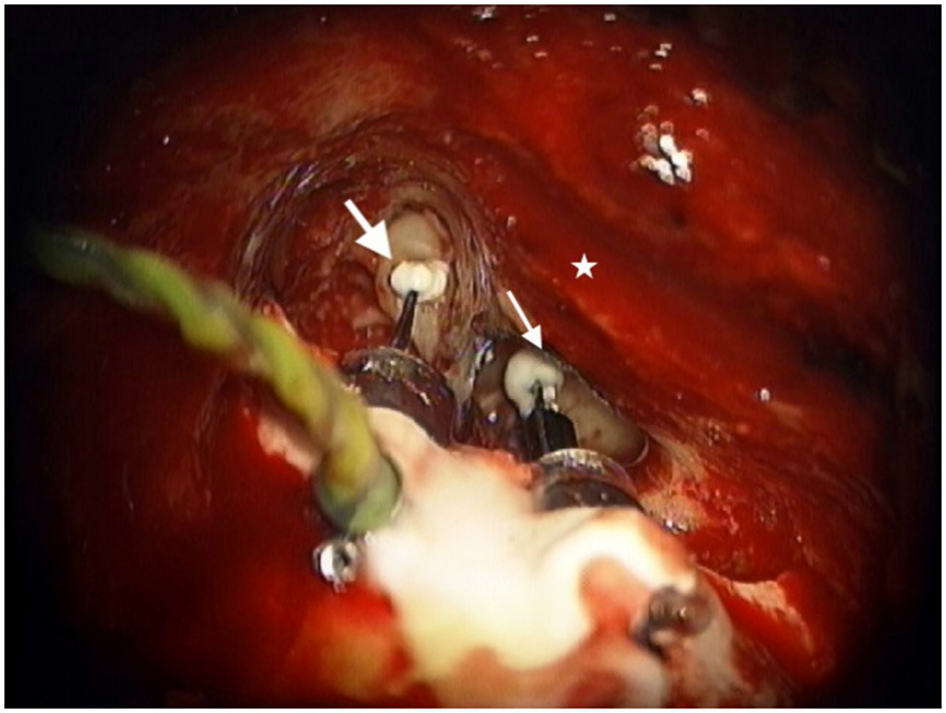
Sensor transducer (large white arrow) and Driver transducer (small white arrow) bodies cemented in mastoid with tips attached to the incus and stapes respectively as viewed through the right-side mastoid exposure. Star represents the posterior external auditory canal.

After ensuring adequate native ossicular movement with laser doppler vibrometry, the incudostapedial joint is disarticulated and the lenticular process of the incus is removed with a laser. The transducers are affixed to the skull using flexible stabilizing bars that facilitate adequate positioning of the sensor transducer with the tip over the incus body and the driver transducer tip to the stapes capitulum. The transducer bodies are affixed to the mastoid using a hydroxyapatite cement and the tips of the transducers are attached to the ossicles using a glass ionomer cement (Figure 1). Measurements are made to ensure adequate ossicular displacement and lack of acoustic feedback before attaching the transducer leads to the SP and closing the incision. Patients are brought back to the clinic for activation approximately 8 weeks after surgery to allow time for the middle ear serous effusion which can impact device function to resolve.

## Outcomes

Ongoing data collection pre and post FDA approval has shown device longevity and programmability over time for proper candidates (13–16). Banakis et al. (17) additionally outlined 16 studies across 18 years showing implantable hearing aid performance benefits. Four of these studies evaluated the Envoy Esteem specifically (11, 16, 18, 19) all of which showed better AMEI objective outcomes compared to CHA as determined by speech recognition scores. Two of these four studies included subjective comparison using the standardized abbreviated profile of hearing aid benefit (APHAB) questionnaire which also showed AMEI preference. These results contrasted with other AMEIs which had more variable outcomes. Common findings amongst Envoy Esteem outcome studies include excellent mid-frequency gain, improved word understanding at conversational levels, and patient preference on validated rating scales. Interestingly, while not the intent of AMEI, one study showed 56.1% of users reported reduction or elimination of tinnitus with the device (14).

Data from 231 ears among these four studies along with extensive phase I and II clinical trial efficacy data (13, 18) demonstrate that AMEIs are no longer experimental; Rather, there is plenty of data showing consistently improved outcomes with AMEI over baseline hearing and CHA treatment. Single-cohort surgeon studies corroborate the importance of surgeon experience to the success of the Esteem recipient, showing declining rates of revision surgery (20) and shorter surgery length (10) over time. During those same multi-year intervals, rates of device explant were fairly stable, between 5 and 11%.

## Candidacy Developments

With a wide candidacy range and variable outcomes, it is reasonable to ask if metrics are available to help predict which patients have the most to gain by changing from CHA to AMEI. One metric that has been proposed as predictive of AMEI advantage is aptly named the speech perception gap (SPgap) by Dyer et al. (21) or earphone to aided difference by McRackan et al. (22). This performance gap describes the difference between a patient's CHA aided speech score at conversational level (i.e., 50 dB HL) and their maximum unaided word recognition score (WRS) already obtained under earphones during the standard audiogram.

**Figure 2:**
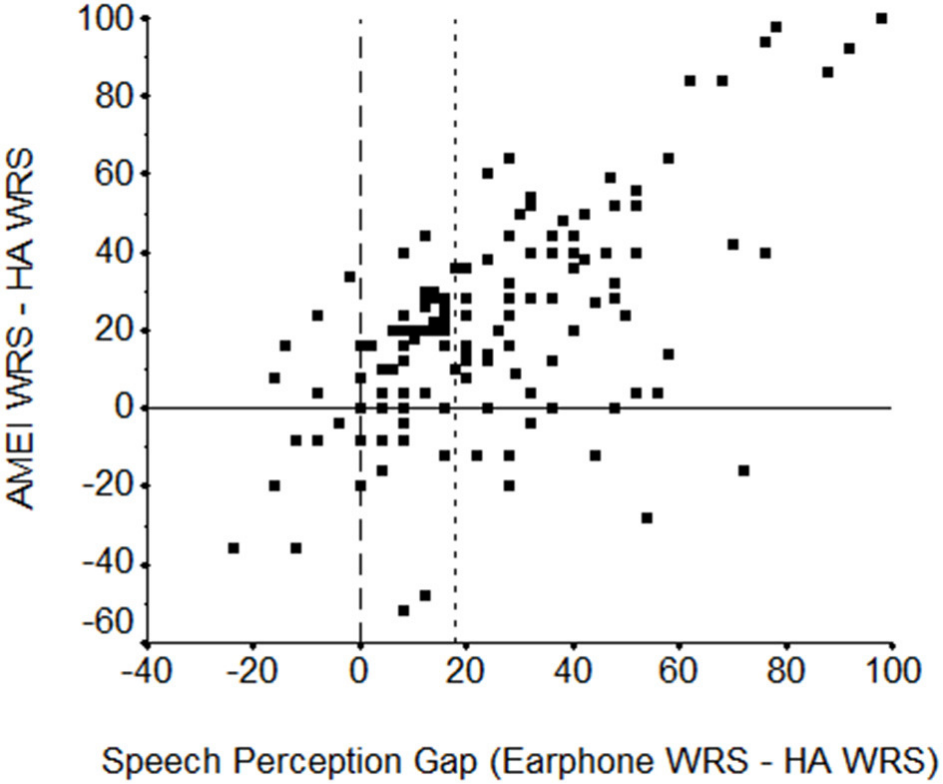
Scatterplot of the earphone (max-WRS) minus hearing aid (HA, at 50 dB HL) word recognition score (WRS) difference in percent correct (“speech perception gap”) by the WRS difference in percent correct between the active middle ear implant (AMEI) and the HA (both at 50 dB HL). The vertical reference line at zero indicates the point of no difference between earphone and HA word recognition scores. To the left, the HA performed better while to the right, earphone performance was better. The horizontal reference line indicates the point of no difference between word recognition scores with the AMEI and HA, below which the HA performance was better and above which the AMEI was better. A second vertical reference line at 18 shows the Earphone-HA speech perception gap at and beyond which >85% of subjects perform better with the AMEI than with their HA. From Shohet et al. (9).

In the Shohet et al. (9) retrospective study, 86% of the 133 subjects demonstrated a SPgap pre-operatively with their appropriately fit CHA, reinforcing that CHA users often do not reach their cochlear potential. The average SPgap of CHA users pre-operatively in this study was 24.7% compared to 3.0% once they received their AMEI. Individual data and trends are plotted in Figure 2. Importantly, it was discovered that the larger the pre-operative SPgap, the greater the performance improvement with AMEI over CHA. Similarly, Chang et al. (23) found an average SPgap of 48.2% with CHA vs. 6.6% with a semi-implantable AMEI. All subjects in that study showed smaller SPgaps with their AMEI compared to their previous CHA. Franks and Jacob show that the SPgap is common in patients with moderate to severe hearing loss and suggest that aided speech testing should be integrated into hearing aid verification for all patients, especially those with maximum unaided word recognition between 40 and 70% (24).

The SPgap metric seems to be sensitive to distinct AMEIs audibility advantages, so it is gaining popularity amongst hearing implant manufacturers who argue they can close this performance gap using direct drive properties. It may also be useful to clinicians who want a simple yet sensitive test to identify good audiometric AMEI candidates.

## Programming Developments

Initially, gain prescription for the Esteem required some trial and error, estimating input gain to achieve a certain amount of output aided gain measured in soundfield. Sound processing options have improved since the first generation Esteem setup, and Envoy describes its current sound processing as hybrid: analog signal processing controlled digitally. Also, in 2014 the prescriptive algorithm called SoundFit became available to help clinicians better prescribe Esteem gain based on baseline audiograms. This statistical NAL-RP based formula was developed using data from 540 users' profile settings and correlating gain outcomes. From this prescriptive starting point, clinicians can now fine-tune different program options into the Esteem, just like a CHA. Software developments have also allowed for more transparent device diagnostic testing and parameter programming over time.

## Current Challenges

Widespread adoption of middle ear implants in the United States has met many hurdles some of which have severely limited their adoption. First, and most impactful, has been the absence of Currently Procedural Terminology (CPT) coding that appropriately describes the services being provided which has prevented payment considerations by Centers for Medicare and Medicaid Services (CMS) as well as commercial payers. Second, with limited third-party reimbursement, many surgeons have given up on implanting or even offering these devices to their patients due to device unfamiliarity and discomfort in performing the complex surgical procedure. Lastly, as CHA sound processing technology advances, the perceived sound advantage of direct drive technology over conventional acoustic stimulation through the ear canal may become less perceivable.

## Future Directions

1. Standardize audiometric test metrics pre and post intervention to allow meta-analyses of AMEI compared to CHA. Individual clinics may also use these metrics to help identify good AMEI candidates and change treatment course if CHA are not effective.
2. Develop a validated questionnaire for assessing AMEI hearing aid lifestyle benefit. Amongst the many benefits of fully implantable AMEI, freedom from daily device maintenance and confidence gained from 24/7 hearing is described empirically but not measured.
3. Determine if direct drive AMEIs are a suitable, less invasive alternative to hybrid cochlear implants.
4. Re-examine if digital processing mechanisms could be supported by today's implantable battery technology.
5. Compare modern extended bandwidth hearing devices to AMEI output. While AMEI provided more gain than open-fit CHA in the past (25, 26), this should be revisited with modern feedback cancellation systems and non-conventional hearing aids.
6. Consider integrating SPgap testing into standard hearing aid care to help identify patients that may be better served with an AMEI.

## Discussion

Envoy Esteem totally implantable AMEI device stability and clinical outcomes have been verified through clinical trials and independent analyses for over 20 years to date. Results consistently show aided hearing thresholds similar to and often better than CHA with notable advantages in mid-frequency gain and superior speech understanding at conversational levels. AMEI should be discussed as an alternative treatment to CHA for proper candidates during hearing device consultations. A simple speech testing metric called the SPgap can be incorporated into busy clinics to help identify good audiometric candidates in addition to those who simply cannot or will not wear CHA. While questionnaires show overall satisfaction with Esteem vs. CHA, opportunities remain to further illuminate why patients prefer the lifestyle advantage of a fully implantable AMEI and “absence of a daily reminder of the disability” (Barbara) (11).

## Author Contributions

JS and JB contributed to conception and design of the study. JB wrote the clinical aspects and JS the surgical details of the manuscript. Both authors contributed to manuscript revision, read, and approved the submitted version.

## Conflict of Interest

JS is a member of the Envoy Medical Advisory Board. None of the authors has a financial relationship with Envoy Medical. The remaining author declares that the research was conducted in the absence of any commercial or financial relationships that could be construed as a potential conflict of interest.

## Publisher's Note

All claims expressed in this article are solely those of the authors and do not necessarily represent those of their affiliated organizations, or those of the publisher, the editors and the reviewers. Any product that may be evaluated in this article, or claim that may be made by its manufacturer, is not guaranteed or endorsed by the publisher.
